# Deciphering the correlation between metabolic activity through 18F-FDG-PET/CT and immune landscape in soft-tissue sarcomas: an insight from the NEOSARCOMICS study

**DOI:** 10.1186/s40364-023-00552-y

**Published:** 2024-01-07

**Authors:** Amandine Crombé, Frédéric Bertolo, Lucile Vanhersecke, Jean-Philippe Guegan, Alban Bessede, Raul Perret, François Le Loarer, Vanessa Chaire, Jean-Michel Coindre, Carlo Lucchesi, Antoine Italiano

**Affiliations:** 1 Department of Oncologic Imaging, Comprehensive Cancer Center, Institut Bergonié, Bordeaux, F-33076 France; 2 Department of Musculoskeletal Imaging, Pellegrin University Hospital, Bordeaux, F-33000 France; 3 SARCOTARGET Team, Bordeaux Institute of Oncology (BRIC) INSERM U1312, Bordeaux, F-33076 France; 4 Department of Bioinformatics, Comprehensive Cancer Center, Institut Bergonié, Bordeaux, F-33076 France; 5 Department of Pathology, Comprehensive Cancer Center, Institut Bergonié, Bordeaux, F-33076 France; 6 Explicyte, Bordeaux, France; 7 Department of Medical Oncology, Comprehensive Cancer Center, Institut Bergonié, Bordeaux, F-33076 France

**Keywords:** Soft-tissue sarcoma, ^18^F-FDG-PET/CT, Transcriptomics, Differential gene expression, Immune landscape

## Abstract

Metabolic elevation in soft-tissue sarcomas (STS), as documented with ^18^F-Fluorodeoxyglucose positron emission tomography (^18^F-FDG-PET/CT) has been linked with cell proliferation, higher grade, and lower survivals. However, the recent diagnostic innovations (CINSARC gene-expression signature and tertiary lymphoid structure [TLS]) and therapeutic innovations (immune checkpoint inhibitors [ICIs]) for STS patients underscore the need to re-assess the role of 18F-FDG-PET/CT. Thus, in this correspondence, our objective was to investigate the correlations between STS metabolism as assessed by nuclear imaging, and the immune landscape as estimated by transcriptomics analysis, immunohistochemistry panels, and TLS assessment. Based on a prospective cohort of 85 adult patients with high-grade STS recruited in the NEOSARCOMICS trial (NCT02789384), we identified 3 metabolic groups according to ^18^F-FDG-PET/CT metrics (metabolic-low [60%], -intermediate [15.3%] and high [24.7%]). We found that T-cells CD8 pathway was significantly enriched in metabolic-high STS. Conversely, several pathways involved in antitumor immune response, cell differentiation and cell cycle, were downregulated in extreme metabolic-low STS. Next, multiplex immunofluorescence showed that densities of CD8+, CD14+, CD45+, CD68+, and c-MAF cells were significantly higher in the metabolic-high group compared to the metabolic-low group. Lastly, no association was found between metabolic group and TLS status. Overall, these results suggest that (i) rapidly proliferating and metabolically active STS can instigate a more robust immune response, thereby attracting immune cells such as T cells and macrophages, and (ii) metabolic activity and TLS could independently influence immune responses.

**Supplementary Information:**

The online version contains supplementary material available at 10.1186/s40364-023-00552-y.

**To the Editor**,

The metabolism of glucose in soft-tissue sarcomas (STS), as documented by ^18^F-Fluorodeoxyglucose positron emission tomography (^18^F-FDG-PET/CT), has been linked with cell proliferation, higher grade, histologic response and survival [1–5]. However, the utility of ^18^F-FDG-PET/CT for STS patients is still debated.

The rapid evolution of therapeutic options for STS patients, along with advancements in histological and molecular characterizations, underscores the need to reassess the role of ^18^F-FDG-PET/CT as a tool for prognostic and theranostic imaging. The FNCLCC grading system is challenged by the complexity index in sarcoma (CINSARC) gene-expression signature [6]. Despite the breakthrough brought about by immune checkpoint inhibitors (ICIs), their response rates remain disappointingly low (5–15%) in unselected populations. This emphasizes the imperative to enhance patient stratification [7–9], which might be feasible through the evaluation of tertiary lymphoid structures (TLS) [10, 11].

The relationships between ^18^F-FDG-PET/CT, tumor microenvironment, and sensitivity to ICIs have been explored in diverse cancers, however, those investigations are noticeably absent for STS. Hence, our objective was to scrutinize the correlations between STS metabolism as assessed by nuclear imaging, and the immune landscape as estimated by transcriptomics analysis, immunohistochemistry panels, and TLS assessment, in order to redefine the role of ^18^F-FDG-PET/CT in the current age of immunotherapy.

Eighty five adult patients (median age: 62 years, 43.5% women), with locally-advanced high-grade STS were enrolled in a precision medicine study between October 2016 and January 2021 (NEOSARCOMICS, NCT02789384). Patient characteristics are outlined in Table 1. Methods are detailed in Supplementary Methods.

**Table 1:** Characteristics of the study population (n = 85)

Characteristics	Patients
**Age (years)**	
Mean ± SD	60.9 ± 11.9
Median (range)	62 (28–81)
**Sex**	
Women	37/85 (43.5%)
Men	48/85 (56.5%)
**WHO performance status**	
0	56/85 (65.9%)
1	29/85 (34.1%)
**Tumor location**	
Head and neck	2/85 (2.4%)
Shoulder girdle	9/85 (10.6%)
Pelvic girdle	7/85 (8.2%)
Trunk wall	9/85 (10.6%)
Upper limb	10/85 (11.8%)
Lower limb	48/85 (56.5%)
**Tumor depth**	
Deep	65/85 (76.5%)
Deep and superficial	18/85 (21.2%)
Superficial	2/85 (2.4%)
**Tumor size (mm)**	
Mean ± SD	114 ± 56
Median (range)	99 (32–274)
**Histological type**	
Undifferentiated pleomorphic sarcoma	40/85 (47.1%)
Rhabdomyosarcoma	9/85 (10.6%)
Leiomyosarcoma	7/85 (8.2%)
M-RC/LPS	7/85 (8.2%)
Synovial sarcoma	7/85 (8.2%)
Pleomophic liposarcoma	6/85 (7.1%)
Other undifferentiated sarcomas	4/85 (4.7%)
Angiosarcoma	2/85 (2.4%)
High-risk solitary fibrous tumor	1/85 (1.2%)
MPNST	1/85 (1.2%)
Myxofibrosarcoma	1/85 (1.2%)
**Curative surgery**	
No	11/85 (12.9%)
Yes	74/85 (87.1%)

NOTE.- Data are number of patients with percentage in parentheses except for age and tumor size. Abbreviations: MPNST: malignant peripheral nerve sheath tumor, M-RC/LPS: myxoid round cells liposarcoma, SD: standard deviation, WHO: World Health Organization.

We extracted maximum, mean, and peak standardized uptake values (SUVs), metabolic tumor volume (MTV), and total lesion glycolysis (TLG) on pre-treatment ^18^F-FDG-PET/CT of each patient. Based on these metrics, we executed an unsupervised classification of the tumors thanks to cross-validated principal component analysis, which identified three metabolic profiles according to their projection on the first principal component (PC1, which was strongly correlated with all PET-related metrics, whereas PC2 was associated with intra-tumoral necrosis and the longest diameter, Supplementary Table T1).

STS with PC1 values falling within the range of the empirical random distribution in over 95% of the instances were deemed ‘metabolic-intermediate’ (15.3% of the sample). The remaining samples with higher PC1 values (24.7%) were categorized as ‘metabolic-high,’ while those exhibiting negative PC1 values (60%) were classified as ‘metabolic-low’ (Supplementary Figure F1).

Next, we explored the transcriptomic characteristics of the metabolic groups. RNA sequencing was available for 32 patients (21.9% from the metabolic-high group, and 62.5% from the metabolic-low group, Fig. 1.A).

We initially executed differential gene expression (DGE) analysis between the metabolic-low and metabolic-high samples, which identifid 67 differentially expressed genes. The T-cells CD8 pathway, which is crucial for the anticancer immune response and the effectiveness of immunotherapies, was significantly enriched in immune-high STS (Supplementary Table T2).

Observing a high transcriptomic variability within the 20 metabolic-low samples, we selected the 7 most extreme metabolic-low samples based on PC1 (Fig. 1.A), revealing 391 differentially expressed genes (Supplementary Table T3, Fig. 1.B). Gene set enrichment analysis highlighted 173 significantly enriched pathways (Fig. 1.C, Supplementary Table T4). Among these pathways, 13 from the LM22 immuno-genesets were significantly inhibited in the metabolic-low group (Fig. 1.D). Specifically, the ICOS gene, the CD27 gene, the Interferon-G gene, and the CXCL9-10-11/CXCR3 axis were downregulated. Additionally, crucial genes involved in the cell cycle were downregulated in the metabolic-low group, most notably: E2F1, CDKN2A and CCNB1. No association was identified between the metabolic groups and CINSARC (*P* = 0.176, Fig. 1.E).

**Fig. 1:**
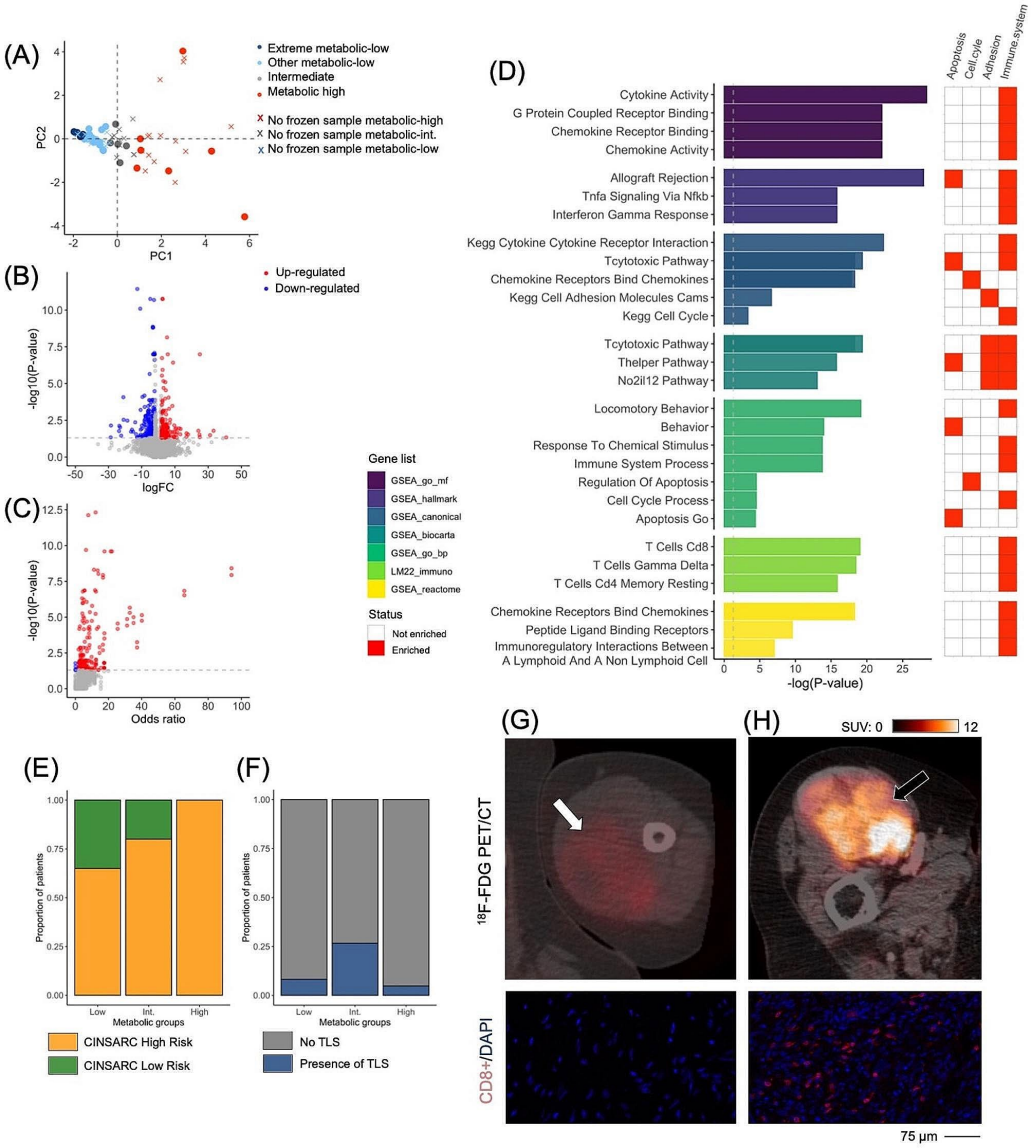
Metabolic profiles according to ^18^F-FDG-PET/CT correlate with the immune landscape of STS. (**A**) Distribution of the available frozen samples depending on principal components (PCs) and subsequent metabolic groups. (**B**) Volcano plot for differential gene expression for the 7 extreme metabolic-low versus 7 extreme metabolic-high comparison. (**C**) Volcano plot for pathway analysis for the 7 extreme metabolic-low versus 7 extreme metabolic-high comparison. (**D**) Summary of the main pathways enriched in metabolic-high group. The matrix on the right highlights the main function of those pathways. Associations between the metabolic groups and the CINSARC signature (**E**) and the TLS status (**F**). Examples of patients with metabolic-high and –low sarcomas: (**G**) a 69 years old woman with a 207 mm-long deep-seated high-grade synovial sarcoma of the arm, which was categorized as metabolic-low according to ^18^F-FDG-PET/CT (SUV_max_=5.1, white arrows) CD8 immunofluorescence showed almost no staining (CD8 + cell density = 1 mm^2^). (**H**) The opposite example corresponds to a 77 years old woman with a 94 mm-long deep-seated high-grade undifferentiated pleomorphic sarcoma of the thigh, which was categorized as metabolic-high according to ^18^F-FDG PET/CT (SUV_max_=22.4, black arrows). CD8 immunofluorescence showed marked and diffuse staining (CD8 + cell density = 539 mm^− 2^). Both sarcomas were CINSARC high risk. Other abbreviations: CINSARC: complexity index in sarcoma; FC: fold change

We have recently reported that TLS may serve as a relevant strategy to identify STS patients who are more likely to benefit from ICI [11]. The TLS status was available in all patients (n = 85). However, no association was found with the metabolic groups, PC1, PC2, or any raw PET-related metrics (Supplementary Table T5, Fig. 1.F).

Lastly, we aimed to validate the observed differences in immune pathway expression at the protein level. We performed immunohistochemistry panels (c-MAF, CD8, CD14, CD20, CD45, and CD68) on 31 patients (25.8% from the metabolic-high group and 58.1% from the metabolic-low group).

We observed consistent positive correlations between cell densities and tumor metabolism as indicated by PC1 (*P*-value range: 0.0247–0.0499). Average densities of CD8+, CD14+, CD45+, CD68+, and c-MAF cells were significantly higher in the metabolic-high group compared to the metabolic-low group (Supplementary Table T6, Fig. 1.G-H).

The relationship between tumor metabolic activity and immune cell infiltration is multifaceted. Our study sheds light on this complex interaction, revealing that STS tumors with high metabolic activity are associated with heightened immune cell infiltration. This observation may stem from the fact that rapidly proliferating and metabolically active STS tumors can instigate a more robust immune response, thereby attracting immune cells such as T cells and macrophages.

Interestingly, we found no collinearity between metabolic activity and TLS status, suggesting that metabolic activity and TLS could independently influence immune responses, even though this finding should be investigated in the different histological subtypes of STS. Therefore, future studies might consider investigating the combined potential of TLS status and PET/CT imaging as predictors of STS patient responsiveness to ICIs, thereby aiding in the development of more effective treatment strategies [12].

### Electronic supplementary material

Below is the link to the electronic supplementary material.

Supplementary Material 1

Supplementary Material 2

Supplementary Material 3

## Data Availability

The datasets and R scripts generated during and/or analyzed during the current study are not publicly available due to the clinical and confidential nature of the material but can be made available from the corresponding author on reasonable request.

## Abbreviations

^18^F-FDG: ^18^F-Fluorodeoxyglucose
CINSARC: Complexity index in sarcoma
DGE: Differential gene expression
FNCLCC: French ‘*Fédération Nationale des Centres de Lutte Contre le Cancer*’
ICI: Immune checkpoint inhibitor
LD: Longest diameter
MTV: Metabolic tumor volume
OS: Overall survival
PCA: Principal component analysis
PCi: i-th principal component
PET: Positron emission tomography
STS: Soft tissue sarcomas
SUV: Standardized uptake value
TLG: Total lesion glycolysis
TLS: Tertiary lymphoid structure
TME: Tumor micro environment
t-SNE: t-distributed stochastic neighbor embedding
VOI: Volume of interest
WHO-PS: World health organization performance status
