# Supplementary material for: Deciphering the correlation between metabolic activity through 18F-FDG-PET/CT and immune landscape in soft-tissue sarcomas: an insight from the NEOSARCOMICS study
Source: Biomark Res. 2024 Jan 7;12:3. doi: 10.1186/s40364-023-00552-y (PMC10773028; doi:10.1186/s40364-023-00552-y)
Supplement: Supplementary file 3 — Supplementary Material 3 [file 40364_2023_552_MOESM3_ESM.docx]

**SUPPLEMENTARY DATA**

**Supplementary Methods**

**1. Study design**

From October 2016 to January 2021, the NEOSARCOMICS trial (NCT02789384) enrolled 90 consecutive adult patients. All participants were newly diagnosed with locally-advanced, non-metastatic, histopathologically-proven high-grade soft-tissue sarcomas (STS) according to the grading system of the French Federation National des Centres de Lutte Contre le Cancer (FNCLCC) [1]. Each patient required neoadjuvant anthracycline-based chemotherapy as part of their curative treatment plan at our center. The study included only those patients for whom pre-treatment ^18^F-FDG-PET/CT data was available (n = 85).

Data collected for this study included patients' age, sex, World Health Organization performance status (WHO-PS), tumor depth, location, and histological type. We also recorded initial treatments, which included radiotherapy (either neoadjuvant or adjuvant), and whether curative surgery was undertaken. In cases where surgery was performed, the proportion of residual stainable tumor cells (STC) on Hematoxylin Eosin and Saffron (HES) slides was documented by senior pathologists from our center, following previously described methods [2].

This precision medicine study was approved by the Institutional Review Board of Institut Bergonié, sarcoma reference center of Bordeaux, France. All patients gave their written informed consent to participate in the study.

**2. ^18^F-FDG-PET/CT acquisition**

Image acquisitions were conducted using a GEMINI® TF-16 system (Philips Healthcare, Best, The Netherlands). Prior to imaging, all patients had fasted for a minimum of 6 hours and maintained a glucose plasma level below 11 mmol/L. They received an intravenous administration of ^18^F-FDG at a dose of 3 MBq/Kg, followed by a further intravenous administration of 20 mg of furosemide after 20 minutes. During this time, patients remained at rest and were hydrated with 500mL of saline solution. Before image acquisition, patients were instructed to empty their bladder.

The ^18^F-FDG-PET/CT acquisition protocol entailed a whole-body scan, spanning from the base of the skull or the vertex to the thighs, performed exactly 60 minutes after the ^18^F-FDG injection. An unenhanced CT scan, in a craniocaudal direction using 16 rows of detectors, was performed for attenuation correction and localization (3-mm thickness; pitch: 0.81; 120kVp; 300 mA). The PET acquisition consisted of 8-9 contiguous steps, each lasting 1 minute and 30 seconds, with the patients' arms raised. Images were acquired using a 3D iterative row-action maximization-likelihood algorithm.

**3. ^18^F-FDG PET/CT analysis**

Following the scans, images were exported in the DICOM format to the LIFEX freeware (v7.0.0, Saclay, France) [3]. We employed the Metabolic Tumor Volume (MTV) protocol of LIFEx for analysis ([www.lifexsoft.org/index.php/resources/documentation](http://www.lifexsoft.org/index.php/resources/documentation)).

A senior radiologist (A.C.), with five years of experience in sarcoma imaging and blinded to all patient features, performed manual segmentation of the entire tumor, slice-by-slice, to derive the whole tumor volume. Within this volume, the radiologist documented the mean and maximum standardized uptake value (SUV_mean_ and SUV_max_, respectively) of the sarcoma. Additionally, the SUV_peak_ was reported, which corresponds to the average SUV value across all voxels contained within a 1 cm^3^ sphere centered on the voxel with the highest SUV_max_ within the total tumor volume [4].

The radiologist further placed a spherical volume-of-interest (VOI) of 3 cm^3^ in the liver, avoiding the liver borders and vessels, to collect the SUV_mean_ of the liver. This measurement was then used to define a threshold to refine the initial entire sarcoma volume: any voxel with an SUV below 1.5 times the SUV_mean_ of the liver was excluded.

The MTV was subsequently defined as the sum of the residual tumor volumes, expressed in cm^3^, and the total lesion glycolysis (TLG) was calculated as the product of these MTVs and their corresponding SUV_mean_ [4].

Upon completion of these measurements, the following metabolic variables were available for all patients: SUV_mean_, SUV_max_, SUV_peak_, MTV, and TLG.

Finally, the radiologist provided an estimation of the percentage of necrosis across the entire tumor volume (characterized by low, fluid-like density on CT-scan and lack of ^18^F-FDG uptake on PET). This was classified into four categories: less than 10%, 10-49%, 50-74%, and 75% or more.

**4. RNA-sequencing and CINSARC signature**

The transcriptomics analysis was carried out in all patients for whom pre-treatment frozen samples were available. The whole-RNA sequencing process was followed by aligning the generated RNA sequences to the transcriptome. Gene expression was subsequently estimated based on the counts of high-quality sequences aligned per gene. To adjust for potential biases, the gene expression counts were normalized utilizing the Voom method, ensuring reliable and statistically sound results [6].

***Sequencing protocol.***

RNA extraction was carried out using the Rneasy Mini Kit from Qiagen (Qiagen, Venlo, Netherlands). Next Generation Sequencing (NGS) library preparation and NGS sequencing were conducted by Integragen (Integragen, Evry, France).

Quality control measures for RNA samples included: (1) preliminary quantitation and purity assessment using Nanodrop, and (2) sensitive quantitation using Qubit 2.0.

The NGS library was prepared using the NEBNext Ultra II mRNA-Seq Kit from NEB (New England Biolabs, Ipswich, Massachusetts, United States).

For NGS sequencing, RNA-Sequencing was performed on the NovaSeq 6000 platform (Illumina Inc. San Diego, Ca, USA) using the prepared library. The qualified libraries were pooled according to their effective concentration and expected data volume, and subsequently loaded into the Illumina sequencers. The generated paired-end sequences had a length of 100 nucleotides, and the target number of RNA sequence pairs was 65 million per patient.

***Sequencing Alignement.***

Bioinformatics analysis, based on the Hg19/Gh37 version of the human genome, was carried out using sequences produced by Whole Transcriptome Sequencing (RNA-Seq).

Initial pre-alignment and quality control of sequences (.fastQ.gz) was executed using the FastqPairedEndValidator to verify the correct pairing of R1 and R2 sequences (<https://github.com/orionzhou/luffy/blob/master/archive/FastqPairedEndValidator.pl>). We also employed Clumpify to eliminate reads with identical sequences (https://github.com/BioInfoTools/BBMap), and Sickle to trim low-quality sequences at the 5’ and 3’ ends (Sickle - <https://github.com/najoshi/sickle>). The SeqPrep package (SeqPrep - <https://github.com/jstjohn/SeqPrep>) was utilized to remove sequencing adaptors from raw reads, and it also identified the proportion of nucleotide fragments where R1 and R2 paired-end reads overlapped, subsequently merging them into single-end reads.

To avoid double coverage bias due to overlapping R1 and R2 sequences, and to fully utilize these fragments, a custom Python script was developed. This script split the merged reads into new non-overlapping R1 and R2 paired-end reads.

The quality-controlled DNA sequences were then aligned to .bam files using Bowtie2 (1) with the ‘–very-sensitive’ alignment strategy parameter. RNA sequences were aligned using Tophat2 (2) and Bowtie2 (1) on both the UCSC hg19 reference genome and transcriptome. Any remaining PCR duplicate reads in post-alignment .bam files were removed using the MarkDuplicates module of the PicardTools suite (PicardTools - <https://broadinstitute.github.io/picard/>).

***Normalization of Gene Expression data****.*

For the normalization of our sample transcript counts, we employed the Voom method. This technique first transforms count data into log2-counts per million (logCPM). It then estimates the mean-variance relationship and subsequently applies this relationship to compute appropriate observational-level weights.

***CINSARC status****.*

The status of CINSARC was determined using a previously established method [7].

**5. Multiplex immunofluorescence**

All staining procedures were performed on 3-μm paraffin slides using a Ventana Discovery Ultra platform (Ventana; Roche Diagnostics). The staining process involved: CD8 (targeting cytotoxic T-cells), CD14 (targeting macrophages), CD20 (targeting B-cells), CD45 (a tyrosine kinase regulating the antigen receptor of T- and B-cells), CD68 (targeting macrophages and monocytes), and c-MAF (a transcription factor involved in T-cell differentiation).

Stainings were carried out using the RUO Discovery Universal protocol, in accordance with the manufacturer's recommendations. Detection was performed using the OmniMap anti-Rb HRP (760-4311; Ventana) and OmniMap anti-Ms HRP (760-4310; Ventana) kits. Following the staining process, slides were scanned using the PerkinElmer Vectra Polaris system. The entire procedure was executed with a Ventana BenchMark ULTRA system.

**6. Assessment of Tertiary Lymphoid Structure (TLS) status**

All hematoxylin eosin and saffron (HES) slides were evaluated blindly by a pathologist (L.V.) according to a previously validated pathological screening method [8]. Briefly, tertiary lymphoid structures (TLS) were identified when lymphoid aggregates contained 50 or more immune cells on HES, which included CD20+ B-cells and CD3+ T-cells as observed in subsequent multiplex immunofluorescence testing.

Mature TLS (mTLS) could be diagnosed solely based on HES when a germinal center was visible. Otherwise, mTLS had to demonstrate a meshwork of CD23+ follicular dendritic cells (FDC) among the CD20+ B-cells, or isolated CD23+ cells exhibiting appropriate FDC morphology. If CD23+ FDC were not present, the TLS were characterized as immature.

**7. Statistical analysis**

Statistical analyses were conducted using the R programming language (version 4.1.0, Vienna, Austria). All tests were two-sided, and a P-value of less than 0.05 was considered statistically significant.

***Defining the metabolic groups.***

The principal component analysis (PCA) was performed using a robust consensus approach based on cross-validation. The outputs of this analysis included the averaged first two principal components (PC1 and PC2) and a classification of the tumors. The results were then confirmed through the use of t-distributed stochastic neighbor embedding (t-SNE) analysis [9].

Robust PCA was implemented using the five ^18^F-FDG PET/CT variables (SUV_max_, SUV_peak_, SUV_mean_, MTV, and TLG). We conducted PCA using the 'prcomp' function from the 'stats' R package, repeating the analysis 1000 times with leave-one-out of 5 samples. The orientation of the PCA was controlled by adding two artificial constant samples containing the minimum and maximum values observed in the dataset. This assured that the values of the minimum and maximum samples were consistently oriented in the same way at each iteration. The consensus PCA, representing all 1000 epochs, was calculated using the mean values of PC1 and PC2 for each sample.

To enhance the robustness of the extreme groups identified by the resampled PCA, we performed another PCA on 85 random samples. These samples were created from each of the 85 original samples by randomly shuffling their values. Their values were centered and scaled using the global mean and variance of the original samples, respectively. The projection of the random samples on the PC1 axis was used to define the bounds of the random region for each iteration. The original samples falling outside this region were considered as high or low metabolic groups, while those falling within were considered intermediate metabolic samples.

In addition to PCA, we employed an alternative method based on dimensionality reduction, t-SNE, using the 'Rtsne' function from the 'Rtsne' R package. Similar to the PCA, a methodology was applied to cross-validate the t-SNE results and identify random regions in the t-SNE space. The random t-SNE representation of the samples was calculated by applying the t-SNE calculation to a randomly obtained matrix.

Lastly, to understand the PCA outputs, associations were investigated with histotypes, CINSARC, LD, raw PET/CT metrics, and the percentage of necrosis evaluated by radiologists.

***Gene-expression profiles of the metabolic groups.***

Differential gene expression (DGE) and gene set enrichment analyses between the metabolic groups identified by PCA were performed in the following manner:

*Differential Analysis.* For the differential gene expression between the metabolic groups, we utilized the statistical t-test from the 'LIMMA' R package. This test computes the fold change and nominal *P*-values for each gene from raw expression values and the normalization weights produced by the 'VOOM' R package. We adjusted the set of nominal *P*-values using the Benjamini-Hochberg procedure. To distinguish significantly upregulated or downregulated transcripts, we set the P-value threshold cutoff to 0.05 and the fold change to 2.0 [10].

*Gene Set Enrichment Analysis.* Enrichment in biological pathways between RNA-Sequencing groups was assessed based on gene sets from Broad Institute’s Molecular Signature Database (MSigDB) and the CYBERSORT LM22 immuno gene sets [11,12]. The gene-set enrichment was performed as follows: We set the total gene count (UNIVERSE) to the total number of genes for which DGE was calculated (18,399 annotated genes in our study). For each biological pathway, we counted the genes involved in the pathway (IN.GENESET) and those that were not (NOT.IN.GENESET). We also split the UNIVERSE into differentially expressed genes (SIG.DGE) and the complement (NOT.SIG.DGE). From this, we constructed a 2 × 2 contingency matrix holding the count of differentially expressed genes in the pathway (SIG.DGE.IN.GENESET) and the count of genes not differentially expressed in the pathway (NOT.SIG.DGE.IN.GENESET). We then performed the hypergeometric test on this table using the fisher.test function in R. We adjusted the raw *P*-values of all tests for each pathway. We further calculated the odds ratio (OR) based on the contingency table and its 95% confidence interval (CI, results of the fisher.test function R base package). We identified significantly enriched pathways as those with an adjusted *P*-value ≤ 0.01 and a lower bound of their 95% CI not lower than 2. We ranked all ENRICHED-tagged pathways by the count of differentially expressed genes in the pathway (SIG.DGE.IN.GENSET). Furthermore, we wanted to qualitatively assess pathways significantly overexpressed or underexpressed in sample groups. For each pathway and each group, we calculated the ratio between the number of genes that were overexpressed in the pathway and the number of DGE genes in the pathway. A ratio near 0 suggests underexpression in the pathway, while a ratio close to 1 suggests overexpression in the reference group. Ratios close to 0.5 were considered informative of no enrichment in either group.

***Immunofluorescence analysis.***

We verified correlations between cell densities and the corresponding gene expression levels using Spearman tests. Subsequently, we examined the relationships between the outputs of the robust PCA and cell densities. Chi-square tests were used to explore associations between TLS status and metabolic groups. We utilized unpaired Wilcoxon tests to investigate associations between TLS status, robust PCA outputs, and raw PET metrics, following a confirmation of the absence of normality using the Shapiro-Wilk normality test.

***Secondary endpoints.***

To investigate associations between PCA outputs, diffusion-based metrics, and markers of treatment response, we used a combination of tests. For numeric variable pairs, we used the Spearman test. For categorical variable pairs, we employed either Chi-square or Fisher tests. When these were not applicable, we resorted to unpaired t-tests or Wilcoxon tests, or one-way analysis of variance (ANOVA-1, with post-hoc Tukey tests) or Kruskal-Wallis tests (with post-hoc Dunn tests). These were chosen depending on the Shapiro-Wilk normality test results on the distribution of the numerical variable and the number of levels of the categorical variable. We adjusted *P*-values for multiple comparisons using the Benjamini-Hochberg procedure.

**REFERENCES**

1 Trojani M, Contesso G, Coindre JM, Rouesse J, Bui NB, de Mascarel A, *et al.* Soft-tissue sarcomas of adults; study of pathological prognostic variables and definition of a histopathological grading system. *Int J Cancer* 1984;**33**:37–42.

2 Wardelmann E, Haas RL, Bovée JVMG, Terrier P, Lazar A, Messiou C, *et al.* Evaluation of response after neoadjuvant treatment in soft tissue sarcomas; the European Organization for Research and Treatment of Cancer-Soft Tissue and Bone Sarcoma Group (EORTC-STBSG) recommendations for pathological examination and reporting. *Eur J Cancer* 2016;**53**:84–95.

3 Nioche C, Orlhac F, Boughdad S, Reuzé S, Goya-Outi J, Robert C, *et al.* LIFEx: A Freeware for Radiomic Feature Calculation in Multimodality Imaging to Accelerate Advances in the Characterization of Tumor Heterogeneity. *Cancer Res* 2018;**78**:4786–9.

4 Wahl RL, Jacene H, Kasamon Y, Lodge MA. From RECIST to PERCIST: Evolving Considerations for PET response criteria in solid tumors. *J Nucl Med* 2009;**50 Suppl 1**:122S-50S.

5 Eisenhauer EA, Therasse P, Bogaerts J, Schwartz LH, Sargent D, Ford R, *et al.* New response evaluation criteria in solid tumours: revised RECIST guideline (version 1.1). *Eur J Cancer* 2009;**45**:228–47.

6 Law CW, Chen Y, Shi W, Smyth GK. voom: Precision weights unlock linear model analysis tools for RNA-seq read counts. *Genome Biol* 2014;**15**:R29.

7 Lesluyes T, Pérot G, Largeau MR, Brulard C, Lagarde P, Dapremont V, *et al.* RNA sequencing validation of the Complexity INdex in SARComas prognostic signature. *Eur J Cancer* 2016;**57**:104–11.

8 Vanhersecke L, Bougouin A, Crombé A, Brunet M, Sofeu C, Parrens M, *et al.* Standardized Pathology Screening of Mature Tertiary Lymphoid Structures in Cancers. *Lab Invest* 2023;**103**:100063.

9 Maaten L van der, Hinton G. Visualizing Data using t-SNE. *Journal of Machine Learning Research* 2008;**9**:2579–605.

10 Ritchie ME, Phipson B, Wu D, Hu Y, Law CW, Shi W, *et al.* limma powers differential expression analyses for RNA-sequencing and microarray studies. *Nucleic Acids Res* 2015;**43**:e47.

11 Subramanian A, Tamayo P, Mootha VK, Mukherjee S, Ebert BL, Gillette MA, *et al.* Gene set enrichment analysis: a knowledge-based approach for interpreting genome-wide expression profiles. *Proc Natl Acad Sci U S A* 2005;**102**:15545–50.

12 Newman AM, Liu CL, Green MR, Gentles AJ, Feng W, Xu Y, *et al.* Robust enumeration of cell subsets from tissue expression profiles. *Nat Methods* 2015;**12**:453–7.

**Supplementary Figure F1.** ^18^F-FDG-PET/CT of patients with extreme values on PC1 and PC2. Tumor location are always shown with black arrowheads. (**A-D**) Patients with the four lowest PC1 values corresponding to the least metabolic tumors. (**E-H**) Patients from the metabolic high group with the four lowest PC2 values corresponding to mid-size tumor with almost no necrosis on imaging. (**I-L**) Patients from the metabolic high group with the four highest PC2 values corresponding to very large tumors with vase areas of necrosis on imaging. Of note, patients form the metabolic low group had PC2 values close to 0. (**M**) Representation of the 12 patients on the PC1 × PC2 coordinates system.

**
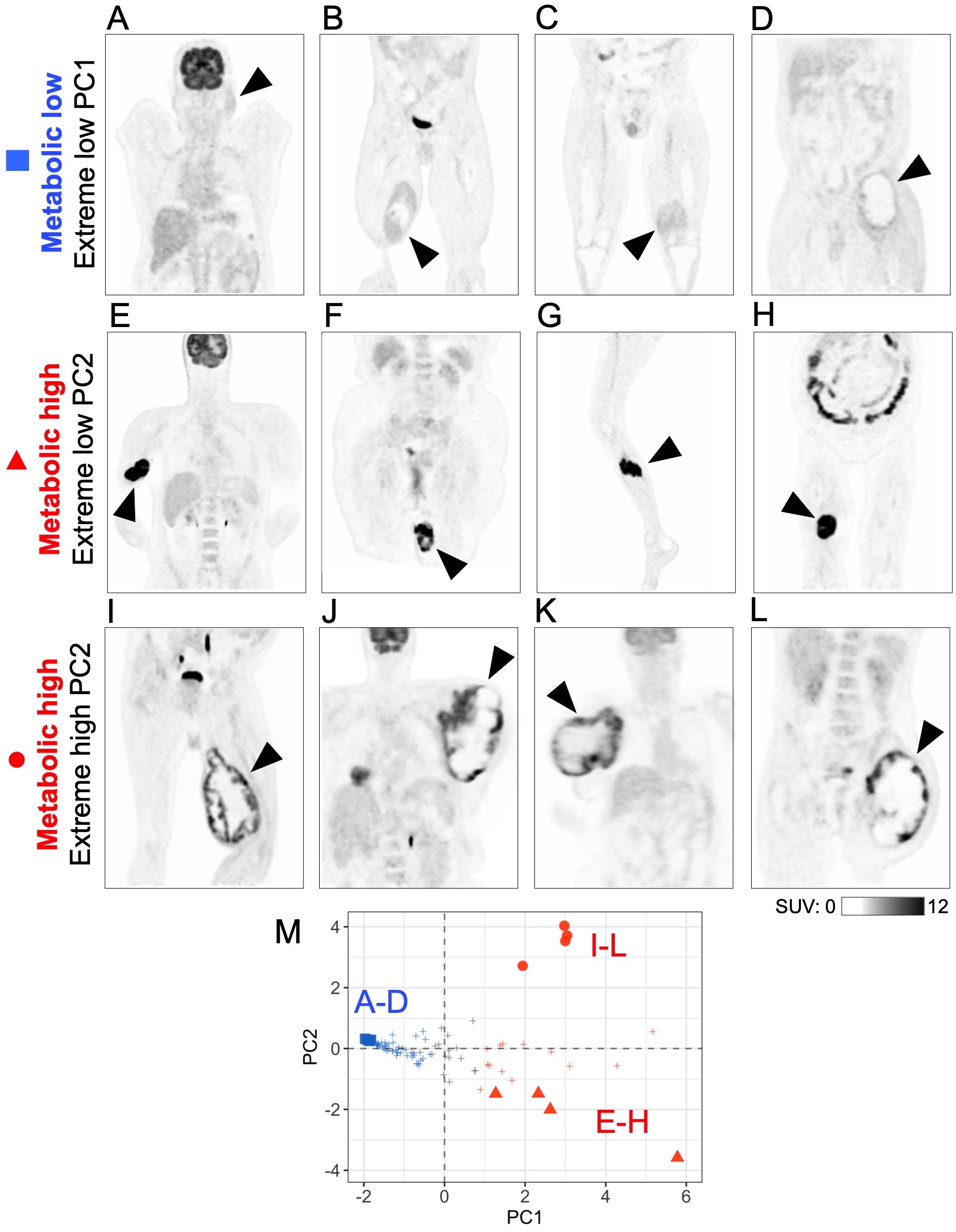
**

**Supplementary Table T1.** Correlations between the average PC1 and PC2 (obtained with cross-validated principal component analysis) and pre-treatment ^18^F-FDG-PET/CT-related metrics

| **PC** | **^18^F-FDG PET metrics** | **Spearman rho** | **P-value** |
| --- | --- | --- | --- |
| **Average PC1** | SUV_mean_ | 0.835 | **<0.0001***** |
|  | SUV_max_ | 0.963 | **<0.0001***** |
|  | SUV_peak_ | 0.973 | **<0.0001***** |
|  | MTV | 0.867 | **<0.0001***** |
|  | TLG | 0.934 | **<0.0001***** |
| **Average PC2** | SUV_mean_ | -0.636 | **<0.0001***** |
|  | SUV_max_ | -0.440 | **<0.0001***** |
|  | SUV_peak_ | -0.445 | **<0.0001***** |
|  | MTV | 0.032 | 0.7701 |
|  | TLG | -0.085 | 0.4410 |

NOTE.- Other abbreviations: MTV: metabolic tumor volume, PC: principal component, SUV: standardized uptake value, TLG: total lesion glycolysis.

***: P < 0.001. Significant results are in bold.

**Supplementary Table T2.** Differential gene expression for the comparison of the 7 available metabolic-high (named A) samples versus the 20 available metabolic-low (named B) samples.

See: SuppTable_T2_DGE_20low-vs-7high.xlsx

**Supplementary Table T3.** Differential gene expression for the comparison of the 7 available metabolic-high samples (named A) versus the 7 extreme metabolic-low samples (i.e., with the lowest PC1 values, named B).

See: SuppTable_T3_DGE_7xlow-vs-7high.xlsx

**Supplementary Table T4.** Pathway analysis for the comparison of the 7 available metabolic-high samples versus the 7 extreme metabolic-low samples (i.e., with the lowest PC1 values). Only significantly enriched pathways (*P* < 0.05) with ratio between the numbers of over-expressed genes in the pathway and the numbers of DGE genes in the pathway ≤ 0.25 (green) and ≥ 0.75 (red) are shown (§).

| ***Genelist*** | ***Geneset*** | ***Adjusted P-value*** | ***Significant DGE in geneset*** | ***Significant DGE not in geneset*** | ***Not significant DGE in geneset*** | ***Not significant DGE not in geneset*** | ***Odds ratio (95%CI for significant DGE vs. Not significant DGE)*** | ***Ratio^§^*** |
| --- | --- | --- | --- | --- | --- | --- | --- | --- |
| GSEA_go_mf | CYTOKINE_ACTIVITY | <0.0001 | 22 | 369 | 88 | 17922 | 12.14 (7.16 - 19.79) | 0 |
| GSEA_hallmark | HALLMARK_ALLOGRAFT_REJECTION | <0.0001 | 27 | 364 | 171 | 17839 | 7.74 (4.89 - 11.84) | 0,04 |
| GSEA_canonical | KEGG_CYTOKINE_CYTOKINE_RECEPTOR_INTERACTION | <0.0001 | 29 | 362 | 224 | 17786 | 6.36 (4.1 - 9.54) | 0 |
| GSEA_go_mf | CHEMOKINE_ACTIVITY | <0.0001 | 13 | 378 | 28 | 17982 | 22.09 (10.41 - 44.47) | 0 |
| GSEA_go_mf | CHEMOKINE_RECEPTOR_BINDING | <0.0001 | 13 | 378 | 29 | 17981 | 21.32 (10.08 - 42.65) | 0 |
| GSEA_go_mf | G_PROTEIN_COUPLED_RECEPTOR_BINDING | <0.0001 | 14 | 377 | 38 | 17972 | 17.56 (8.71 - 33.45) | 0 |
| GSEA_biocarta | BIOCARTA_TCYTOTOXIC_PATHWAY | <0.0001 | 8 | 383 | 4 | 18006 | 94.03 (25.01 - 426.48) | 0 |
| GSEA_go_bp | LOCOMOTORY_BEHAVIOR | <0.0001 | 17 | 374 | 73 | 17937 | 11.17 (6.11 - 19.34) | 0 |
| LM22_immuno | T_cells_CD8 | <0.0001 | 13 | 378 | 46 | 17964 | 13.43 (6.6 - 25.55) | 0 |
| LM22_immuno | T_cells_gamma_delta | <0.0001 | 12 | 379 | 41 | 17969 | 13.88 (6.58 - 27.16) | 0 |
| GSEA_canonical | BIOCARTA_TCYTOTOXIC_PATHWAY | <0.0001 | 8 | 383 | 4 | 18006 | 94.03 (25.01 - 426.48) | 0 |
| GSEA_canonical | REACTOME_CHEMOKINE_RECEPTORS_BIND_CHEMOKINES | <0.0001 | 13 | 378 | 37 | 17973 | 16.71 (8.07 - 32.48) | 0 |
| GSEA_reactome | REACTOME_CHEMOKINE_RECEPTORS_BIND_CHEMOKINES | <0.0001 | 13 | 378 | 37 | 17973 | 16.71 (8.07 - 32.48) | 0 |
| LM22_immuno | T_cells_CD4_memory_resting | <0.0001 | 11 | 380 | 44 | 17966 | 11.82 (5.46 - 23.47) | 0 |
| GSEA_hallmark | HALLMARK_INTERFERON_GAMMA_RESPONSE | <0.0001 | 20 | 371 | 175 | 17835 | 5.49 (3.24 - 8.87) | 0 |
| GSEA_hallmark | HALLMARK_TNFA_SIGNALING_VIA_NFKB | <0.0001 | 20 | 371 | 177 | 17833 | 5.43 (3.2 - 8.76) | 0 |
| GSEA_biocarta | BIOCARTA_THELPER_PATHWAY | <0.0001 | 7 | 384 | 5 | 18005 | 65.64 (17.81 - 264.1) | 0 |
| GSEA_canonical | PID_IL12_2PATHWAY | <0.0001 | 13 | 378 | 50 | 17960 | 12.35 (6.1 - 23.31) | 0,08 |
| GSEA_canonical | NABA_SECRETED_FACTORS | <0.0001 | 28 | 363 | 304 | 17706 | 4.49 (2.9 - 6.73) | 0,07 |
| GSEA_canonical | BIOCARTA_THELPER_PATHWAY | <0.0001 | 7 | 384 | 5 | 18005 | 65.64 (17.81 - 264.1) | 0 |
| GSEA_go_mf | RECEPTOR_BINDING | <0.0001 | 28 | 363 | 340 | 17670 | 4.01 (2.59 - 6) | 0,11 |
| GSEA_hallmark | HALLMARK_INFLAMMATORY_RESPONSE | <0.0001 | 19 | 372 | 179 | 17831 | 5.09 (2.96 - 8.29) | 0 |
| GSEA_go_bp | BEHAVIOR | <0.0001 | 18 | 373 | 128 | 17882 | 6.74 (3.83 - 11.23) | 0,06 |
| GSEA_go_bp | RESPONSE_TO_CHEMICAL_STIMULUS | <0.0001 | 25 | 366 | 271 | 17739 | 4.47 (2.81 - 6.85) | 0,04 |
| GSEA_go_bp | IMMUNE_SYSTEM_PROCESS | <0.0001 | 26 | 365 | 289 | 17721 | 4.37 (2.77 - 6.64) | 0,04 |
| LM22_immuno | T_cells_regulatory_.Tregs. | <0.0001 | 10 | 381 | 47 | 17963 | 10.03 (4.48 - 20.3) | 0 |
| GSEA_biocarta | BIOCARTA_NO2IL12_PATHWAY | <0.0001 | 7 | 384 | 10 | 18000 | 32.81 (10.53 - 95.93) | 0,14 |
| GSEA_canonical | KEGG_HEMATOPOIETIC_CELL_LINEAGE | <0.0001 | 13 | 378 | 71 | 17939 | 8.69 (4.37 - 15.99) | 0 |
| GSEA_canonical | BIOCARTA_NO2IL12_PATHWAY | <0.0001 | 7 | 384 | 10 | 18000 | 32.81 (10.53 - 95.93) | 0,14 |
| GSEA_go_bp | RESPONSE_TO_EXTERNAL_STIMULUS | <0.0001 | 24 | 367 | 278 | 17732 | 4.17 (2.59 - 6.43) | 0,04 |
| LM22_immuno | T_cells_CD4_memory_activated | <0.0001 | 8 | 383 | 31 | 17979 | 12.11 (4.78 - 27.15) | 0 |
| GSEA_biocarta | BIOCARTA_CTL_PATHWAY | <0.0001 | 6 | 385 | 7 | 18003 | 40.08 (11.06 - 139.77) | 0 |
| GSEA_go_bp | IMMUNE_RESPONSE | <0.0001 | 20 | 371 | 203 | 17807 | 4.73 (2.8 - 7.6) | 0,05 |
| GSEA_biocarta | BIOCARTA_GRANULOCYTES_PATHWAY | <0.0001 | 6 | 385 | 8 | 18002 | 35.07 (9.97 - 115.86) | 0 |
| GSEA_canonical | KEGG_CHEMOKINE_SIGNALING_PATHWAY | <0.0001 | 18 | 373 | 166 | 17844 | 5.19 (2.97 - 8.57) | 0,06 |
| GSEA_biocarta | BIOCARTA_IL17_PATHWAY | <0.0001 | 6 | 385 | 9 | 18001 | 31.17 (9.07 - 98.57) | 0 |
| GSEA_canonical | BIOCARTA_CTL_PATHWAY | <0.0001 | 6 | 385 | 7 | 18003 | 40.08 (11.06 - 139.77) | 0 |
| GSEA_go_bp | DEFENSE_RESPONSE | <0.0001 | 21 | 370 | 236 | 17774 | 4.27 (2.57 - 6.78) | 0,05 |
| LM22_immuno | T_cells_CD4_naive | <0.0001 | 8 | 383 | 38 | 17972 | 9.88 (3.95 - 21.67) | 0,12 |
| GSEA_hallmark | HALLMARK_IL6_JAK_STAT3_SIGNALING | <0.0001 | 11 | 380 | 75 | 17935 | 6.92 (3.29 - 13.24) | 0 |
| GSEA_canonical | REACTOME_PEPTIDE_LIGAND_BINDING_RECEPTORS | <0.0001 | 17 | 374 | 158 | 17852 | 5.14 (2.89 - 8.6) | 0,12 |
| GSEA_canonical | BIOCARTA_GRANULOCYTES_PATHWAY | <0.0001 | 6 | 385 | 8 | 18002 | 35.07 (9.97 - 115.86) | 0 |
| GSEA_biocarta | BIOCARTA_LAIR_PATHWAY | <0.0001 | 6 | 385 | 11 | 17999 | 25.5 (7.7 - 75.64) | 0 |
| GSEA_canonical | BIOCARTA_IL17_PATHWAY | <0.0001 | 6 | 385 | 9 | 18001 | 31.17 (9.07 - 98.57) | 0 |
| GSEA_canonical | NABA_MATRISOME_ASSOCIATED | <0.0001 | 38 | 353 | 691 | 17319 | 2.7 (1.86 - 3.81) | 0,11 |
| LM22_immuno | Dendritic_cells_activated | <0.0001 | 8 | 383 | 45 | 17965 | 8.34 (3.37 - 18.03) | 0 |
| GSEA_canonical | KEGG_PRIMARY_IMMUNODEFICIENCY | <0.0001 | 8 | 383 | 27 | 17983 | 13.91 (5.42 - 31.72) | 0,12 |
| LM22_immuno | NK_cells_activated | 0.0001 | 8 | 383 | 48 | 17962 | 7.82 (3.17 - 16.81) | 0 |
| GSEA_canonical | NABA_MATRISOME | 0.0001 | 46 | 345 | 956 | 17054 | 2.38 (1.7 - 3.27) | 0,22 |
| GSEA_canonical | BIOCARTA_LAIR_PATHWAY | 0.0001 | 6 | 385 | 11 | 17999 | 25.5 (7.7 - 75.64) | 0 |
| GSEA_canonical | PID_IL23_PATHWAY | 0.0001 | 8 | 383 | 29 | 17981 | 12.95 (5.08 - 29.26) | 0,12 |
| GSEA_reactome | REACTOME_PEPTIDE_LIGAND_BINDING_RECEPTORS | 0.0001 | 17 | 374 | 158 | 17852 | 5.14 (2.89 - 8.6) | 0,12 |
| GSEA_canonical | PID_CD8_TCR_DOWNSTREAM_PATHWAY | 0.0001 | 10 | 381 | 55 | 17955 | 8.57 (3.86 - 17.13) | 0 |
| GSEA_biocarta | BIOCARTA_DC_PATHWAY | 0.0001 | 6 | 385 | 16 | 17994 | 17.53 (5.58 - 47.45) | 0 |
| LM22_immuno | Macrophages_M1 | 0.0001 | 7 | 384 | 40 | 17970 | 8.19 (3.07 - 18.63) | 0 |
| GSEA_canonical | REACTOME_IMMUNOREGULATORY_INTERACTIONS_BETWEEN_A_LYMPHOID_AND_A_NON_LYMPHOID_CELL | 0.0003 | 9 | 382 | 50 | 17960 | 8.46 (3.63 - 17.54) | 0 |
| LM22_immuno | NK_cells_resting | 0.0003 | 7 | 384 | 46 | 17964 | 7.12 (2.69 - 16.01) | 0 |
| GSEA_canonical | BIOCARTA_DC_PATHWAY | 0.0003 | 6 | 385 | 16 | 17994 | 17.53 (5.58 - 47.45) | 0 |
| GSEA_go_bp | RESPONSE_TO_BIOTIC_STIMULUS | 0.0005 | 12 | 379 | 101 | 17909 | 5.61 (2.78 - 10.35) | 0,17 |
| GSEA_biocarta | BIOCARTA_TCAPOPTOSIS_PATHWAY | 0.0006 | 4 | 387 | 5 | 18005 | 37.22 (7.35 - 173.6) | 0 |
| GSEA_biocarta | BIOCARTA_STATHMIN_PATHWAY | 0.0008 | 5 | 386 | 14 | 17996 | 16.65 (4.67 - 49.23) | 0 |
| GSEA_reactome | REACTOME_IMMUNOREGULATORY_INTERACTIONS_BETWEEN_A_LYMPHOID_AND_A_NON_LYMPHOID_CELL | 0.0009 | 9 | 382 | 50 | 17960 | 8.46 (3.63 - 17.54) | 0 |
| GSEA_biocarta | BIOCARTA_IL12_PATHWAY | 0.0013 | 5 | 386 | 16 | 17994 | 14.57 (4.15 - 41.91) | 0 |
| GSEA_canonical | BIOCARTA_TCAPOPTOSIS_PATHWAY | 0.0013 | 4 | 387 | 5 | 18005 | 37.22 (7.35 - 173.6) | 0 |
| GSEA_canonical | KEGG_CELL_ADHESION_MOLECULES_CAMS | 0.0013 | 12 | 379 | 119 | 17891 | 4.76 (2.37 - 8.72) | 0 |
| LM22_immuno | T_cells_follicular_helper | 0.0014 | 6 | 385 | 45 | 17965 | 6.22 (2.16 - 14.74) | 0 |
| GSEA_go_bp | CELLULAR_DEFENSE_RESPONSE | 0.0014 | 8 | 383 | 46 | 17964 | 8.16 (3.3 - 17.6) | 0 |
| GSEA_go_bp | SIGNAL_TRANSDUCTION | 0.0018 | 59 | 332 | 1533 | 16477 | 1.91 (1.42 - 2.54) | 0,15 |
| GSEA_canonical | BIOCARTA_STATHMIN_PATHWAY | 0.002 | 5 | 386 | 14 | 17996 | 16.65 (4.67 - 49.23) | 0 |
| GSEA_hallmark | HALLMARK_COMPLEMENT | 0.0021 | 13 | 378 | 180 | 17830 | 3.41 (1.76 - 6.04) | 0 |
| GSEA_canonical | KEGG_NOD_LIKE_RECEPTOR_SIGNALING_PATHWAY | 0.0025 | 8 | 383 | 54 | 17956 | 6.95 (2.83 - 14.81) | 0 |
| GSEA_canonical | BIOCARTA_IL12_PATHWAY | 0.0032 | 5 | 386 | 16 | 17994 | 14.57 (4.15 - 41.91) | 0 |
| GSEA_canonical | KEGG_JAK_STAT_SIGNALING_PATHWAY | 0.0042 | 12 | 379 | 138 | 17872 | 4.1 (2.05 - 7.48) | 0 |
| GSEA_canonical | KEGG_T_CELL_RECEPTOR_SIGNALING_PATHWAY | 0.0043 | 10 | 381 | 97 | 17913 | 4.85 (2.23 - 9.39) | 0 |
| GSEA_hallmark | HALLMARK_COAGULATION | 0.0045 | 10 | 381 | 125 | 17885 | 3.76 (1.74 - 7.21) | 0 |
| GSEA_go_bp | INFLAMMATORY_RESPONSE | 0.0051 | 11 | 380 | 114 | 17896 | 4.54 (2.19 - 8.53) | 0 |
| GSEA_canonical | REACTOME_CLASS_A1_RHODOPSIN_LIKE_RECEPTORS | 0.0051 | 17 | 374 | 264 | 17746 | 3.06 (1.73 - 5.05) | 0,12 |
| GSEA_biocarta | BIOCARTA_NKT_PATHWAY | 0.006 | 5 | 386 | 24 | 17986 | 9.71 (2.88 - 26.13) | 0 |
| GSEA_canonical | REACTOME_CYTOKINE_SIGNALING_IN_IMMUNE_SYSTEM | 0.0067 | 16 | 375 | 245 | 17765 | 3.09 (1.72 - 5.19) | 0 |
| GSEA_go_bp | MULTI_ORGANISM_PROCESS | 0.0092 | 12 | 379 | 146 | 17864 | 3.87 (1.94 - 7.05) | 0,08 |
| GSEA_biocarta | BIOCARTA_TOB1_PATHWAY | 0.0094 | 4 | 387 | 15 | 17995 | 12.4 (2.98 - 39.14) | 0 |
| GSEA_biocarta | BIOCARTA_CTLA4_PATHWAY | 0.0094 | 4 | 387 | 15 | 17995 | 12.4 (2.98 - 39.14) | 0 |
| GSEA_canonical | REACTOME_INTERFERON_GAMMA_SIGNALING | 0.0102 | 7 | 384 | 52 | 17958 | 6.3 (2.4 - 14.03) | 0 |
| GSEA_go_bp | VIRAL_REPRODUCTION | 0.0109 | 6 | 385 | 34 | 17976 | 8.24 (2.81 - 20) | 0 |
| GSEA_go_bp | T_CELL_ACTIVATION | 0.0109 | 6 | 385 | 37 | 17973 | 7.57 (2.6 - 18.24) | 0 |
| GSEA_go_bp | LYMPHOCYTE_ACTIVATION | 0.0109 | 7 | 384 | 52 | 17958 | 6.3 (2.4 - 14.03) | 0 |
| GSEA_go_bp | RESPONSE_TO_OTHER_ORGANISM | 0.0109 | 8 | 383 | 70 | 17940 | 5.35 (2.21 - 11.24) | 0,12 |
| GSEA_go_bp | CELL_CYCLE_PHASE | 0.0109 | 12 | 379 | 157 | 17853 | 3.6 (1.81 - 6.54) | 0,08 |
| GSEA_go_bp | CELL_CYCLE_PROCESS | 0.0109 | 13 | 378 | 179 | 17831 | 3.43 (1.77 - 6.08) | 0,08 |
| GSEA_go_bp | REGULATION_OF_APOPTOSIS | 0.0109 | 18 | 373 | 310 | 17700 | 2.76 (1.59 - 4.49) | 0,11 |
| GSEA_go_bp | REGULATION_OF_PROGRAMMED_CELL_DEATH | 0.0109 | 18 | 373 | 311 | 17699 | 2.75 (1.59 - 4.47) | 0,11 |
| GSEA_canonical | KEGG_TOLL_LIKE_RECEPTOR_SIGNALING_PATHWAY | 0.0121 | 9 | 382 | 92 | 17918 | 4.59 (2.02 - 9.19) | 0 |
| GSEA_go_bp | INTERPHASE_OF_MITOTIC_CELL_CYCLE | 0.0123 | 7 | 384 | 55 | 17955 | 5.95 (2.27 - 13.21) | 0 |
| GSEA_go_bp | APOPTOSIS_GO | 0.0123 | 21 | 370 | 396 | 17614 | 2.52 (1.53 - 3.97) | 0,1 |
| GSEA_go_bp | PROGRAMMED_CELL_DEATH | 0.0123 | 21 | 370 | 397 | 17613 | 2.52 (1.52 - 3.96) | 0,1 |
| GSEA_go_bp | RESPONSE_TO_STRESS | 0.0123 | 23 | 368 | 466 | 17544 | 2.35 (1.46 - 3.63) | 0,13 |
| GSEA_canonical | BIOCARTA_NKT_PATHWAY | 0.0129 | 5 | 386 | 24 | 17986 | 9.71 (2.88 - 26.13) | 0 |
| GSEA_go_cc | EXTRACELLULAR_SPACE | 0.0129 | 16 | 375 | 223 | 17787 | 3.4 (1.89 - 5.72) | 0 |
| LM22_immuno | Mast_cells_activated | 0.013 | 4 | 387 | 33 | 17977 | 5.63 (1.44 - 15.94) | 0 |
| LM22_immuno | Neutrophils | 0.013 | 5 | 386 | 53 | 17957 | 4.39 (1.36 - 10.98) | 0 |
| GSEA_canonical | REACTOME_IMMUNE_SYSTEM | 0.0149 | 35 | 356 | 836 | 17174 | 2.02 (1.37 - 2.89) | 0 |
| GSEA_go_bp | CELL_DEVELOPMENT | 0.0154 | 25 | 366 | 537 | 17473 | 2.22 (1.41 - 3.37) | 0,16 |
| GSEA_reactome | REACTOME_CLASS_A1_RHODOPSIN_LIKE_RECEPTORS | 0.0155 | 17 | 374 | 264 | 17746 | 3.06 (1.73 - 5.05) | 0,12 |
| GSEA_biocarta | BIOCARTA_LYM_PATHWAY | 0.0158 | 3 | 388 | 8 | 18002 | 17.4 (2.96 - 72.76) | 0 |
| GSEA_biocarta | BIOCARTA_BLYMPHOCYTE_PATHWAY | 0.0158 | 3 | 388 | 8 | 18002 | 17.4 (2.96 - 72.76) | 0 |
| GSEA_biocarta | BIOCARTA_TCRA_PATHWAY | 0.0158 | 3 | 388 | 8 | 18002 | 17.4 (2.96 - 72.76) | 0 |
| GSEA_biocarta | BIOCARTA_MONOCYTE_PATHWAY | 0.0158 | 3 | 388 | 8 | 18002 | 17.4 (2.96 - 72.76) | 0 |
| GSEA_biocarta | BIOCARTA_CELLCYCLE_PATHWAY | 0.0158 | 4 | 387 | 19 | 17991 | 9.79 (2.41 - 29.63) | 0 |
| GSEA_go_bp | LEUKOCYTE_ACTIVATION | 0.0159 | 7 | 384 | 60 | 17950 | 5.45 (2.09 - 12.04) | 0 |
| GSEA_go_bp | ESTABLISHMENT_AND_OR_MAINTENANCE_OF_CELL_POLARITY | 0.0159 | 4 | 387 | 15 | 17995 | 12.4 (2.98 - 39.14) | 0,25 |
| GSEA_go_bp | INTERPHASE | 0.0159 | 7 | 384 | 61 | 17949 | 5.36 (2.06 - 11.84) | 0 |
| GSEA_go_bp | RESPONSE_TO_WOUNDING | 0.0159 | 12 | 379 | 173 | 17837 | 3.26 (1.64 - 5.92) | 0 |
| GSEA_go_bp | REGULATION_OF_CELL_PROLIFERATION | 0.0159 | 16 | 375 | 279 | 17731 | 2.71 (1.51 - 4.54) | 0,19 |
| GSEA_canonical | PID_NFAT_TFPATHWAY | 0.0164 | 6 | 385 | 41 | 17969 | 6.83 (2.35 - 16.31) | 0 |
| GSEA_reactome | REACTOME_CYTOKINE_SIGNALING_IN_IMMUNE_SYSTEM | 0.0175 | 16 | 375 | 245 | 17765 | 3.09 (1.72 - 5.19) | 0 |
| GSEA_go_bp | VIRAL_GENOME_REPLICATION | 0.0186 | 4 | 387 | 16 | 17994 | 11.62 (2.81 - 36.22) | 0 |
| GSEA_canonical | BIOCARTA_TOB1_PATHWAY | 0.0206 | 4 | 387 | 15 | 17995 | 12.4 (2.98 - 39.14) | 0 |
| GSEA_canonical | BIOCARTA_CTLA4_PATHWAY | 0.0206 | 4 | 387 | 15 | 17995 | 12.4 (2.98 - 39.14) | 0 |
| GSEA_canonical | REACTOME_HEMOSTASIS | 0.0227 | 21 | 370 | 426 | 17584 | 2.34 (1.42 - 3.68) | 0,19 |
| GSEA_canonical | PID_AP1_PATHWAY | 0.0228 | 7 | 384 | 63 | 17947 | 5.19 (1.99 - 11.44) | 0,14 |
| GSEA_hallmark | HALLMARK_INTERFERON_ALPHA_RESPONSE | 0.0229 | 7 | 384 | 86 | 17924 | 3.8 (1.47 - 8.24) | 0 |
| GSEA_reactome | REACTOME_INTERFERON_GAMMA_SIGNALING | 0.0235 | 7 | 384 | 52 | 17958 | 6.3 (2.4 - 14.03) | 0 |
| GSEA_go_bp | CELL_ACTIVATION | 0.0238 | 7 | 384 | 67 | 17943 | 4.88 (1.88 - 10.72) | 0 |
| GSEA_go_bp | REGULATION_OF_MULTICELLULAR_ORGANISMAL_PROCESS | 0.0269 | 10 | 381 | 136 | 17874 | 3.45 (1.6 - 6.61) | 0,1 |
| GSEA_canonical | PID_CD8_TCR_PATHWAY | 0.0273 | 6 | 385 | 47 | 17963 | 5.96 (2.07 - 14.08) | 0 |
| GSEA_go_bp | CELL_PROLIFERATION_GO_0008283 | 0.0275 | 22 | 369 | 472 | 17538 | 2.22 (1.36 - 3.45) | 0,18 |
| GSEA_go_bp | REGULATION_OF_MOLECULAR_FUNCTION | 0.0275 | 16 | 375 | 300 | 17710 | 2.52 (1.41 - 4.21) | 0,25 |
| GSEA_canonical | PID_FRA_PATHWAY | 0.028 | 5 | 386 | 31 | 17979 | 7.51 (2.27 - 19.62) | 0 |
| GSEA_go_bp | REGULATION_OF_TRANSCRIPTION_FACTOR_ACTIVITY | 0.0284 | 5 | 386 | 34 | 17976 | 6.85 (2.08 - 17.74) | 0 |
| GSEA_reactome | REACTOME_IMMUNE_SYSTEM | 0.033 | 35 | 356 | 836 | 17174 | 2.02 (1.37 - 2.89) | 0 |
| GSEA_go_bp | MITOTIC_CELL_CYCLE | 0.0336 | 10 | 381 | 143 | 17867 | 3.28 (1.53 - 6.27) | 0 |
| GSEA_go_bp | REGULATION_OF_MITOSIS | 0.0339 | 5 | 386 | 36 | 17974 | 6.47 (1.97 - 16.66) | 0 |
| GSEA_biocarta | BIOCARTA_INFLAM_PATHWAY | 0.034 | 4 | 387 | 25 | 17985 | 7.44 (1.87 - 21.67) | 0 |
| GSEA_go_cc | EXTERNAL_SIDE_OF_PLASMA_MEMBRANE | 0.0348 | 4 | 387 | 12 | 17998 | 15.5 (3.63 - 51.33) | 0 |
| GSEA_go_cc | EXTRACELLULAR_REGION | 0.0348 | 21 | 370 | 415 | 17595 | 2.41 (1.46 - 3.78) | 0,05 |
| GSEA_go_cc | PLASMA_MEMBRANE | 0.0348 | 48 | 343 | 1322 | 16688 | 1.77 (1.27 - 2.41) | 0,25 |
| GSEA_biocarta | BIOCARTA_ERYTH_PATHWAY | 0.0353 | 3 | 388 | 12 | 17998 | 11.6 (2.09 - 43.19) | 0 |
| GSEA_biocarta | BIOCARTA_STEM_PATHWAY | 0.0353 | 3 | 388 | 12 | 17998 | 11.6 (2.09 - 43.19) | 0 |
| GSEA_canonical | BIOCARTA_LYM_PATHWAY | 0.0354 | 3 | 388 | 8 | 18002 | 17.4 (2.96 - 72.76) | 0 |
| GSEA_canonical | BIOCARTA_BLYMPHOCYTE_PATHWAY | 0.0354 | 3 | 388 | 8 | 18002 | 17.4 (2.96 - 72.76) | 0 |
| GSEA_canonical | BIOCARTA_TCRA_PATHWAY | 0.0354 | 3 | 388 | 8 | 18002 | 17.4 (2.96 - 72.76) | 0 |
| GSEA_canonical | BIOCARTA_MONOCYTE_PATHWAY | 0.0354 | 3 | 388 | 8 | 18002 | 17.4 (2.96 - 72.76) | 0 |
| GSEA_canonical | ST_STAT3_PATHWAY | 0.0354 | 3 | 388 | 8 | 18002 | 17.4 (2.96 - 72.76) | 0 |
| GSEA_canonical | BIOCARTA_CELLCYCLE_PATHWAY | 0.0354 | 4 | 387 | 19 | 17991 | 9.79 (2.41 - 29.63) | 0 |
| GSEA_canonical | KEGG_ANTIGEN_PROCESSING_AND_PRESENTATION | 0.0354 | 7 | 384 | 71 | 17939 | 4.61 (1.78 - 10.08) | 0 |
| GSEA_canonical | KEGG_CELL_CYCLE | 0.0354 | 9 | 382 | 115 | 17895 | 3.67 (1.62 - 7.28) | 0,11 |
| GSEA_canonical | PID_SHP2_PATHWAY | 0.0354 | 6 | 385 | 52 | 17958 | 5.38 (1.88 - 12.63) | 0,17 |
| GSEA_go_cc | EXTRACELLULAR_REGION_PART | 0.0357 | 17 | 374 | 314 | 17696 | 2.56 (1.46 - 4.22) | 0,06 |
| GSEA_go_mf | RECEPTOR_ACTIVITY | 0.0384 | 25 | 366 | 544 | 17466 | 2.19 (1.39 - 3.32) | 0,2 |
| GSEA_canonical | PID_AMB2_NEUTROPHILS_PATHWAY | 0.0415 | 5 | 386 | 36 | 17974 | 6.47 (1.97 - 16.66) | 0 |
| GSEA_hallmark | HALLMARK_SPERMATOGENESIS | 0.0421 | 8 | 383 | 125 | 17885 | 2.99 (1.25 - 6.13) | 0,25 |
| GSEA_canonical | PID_INTEGRIN_CS_PATHWAY | 0.0423 | 4 | 387 | 21 | 17989 | 8.85 (2.2 - 26.4) | 0 |
| GSEA_canonical | KEGG_NATURAL_KILLER_CELL_MEDIATED_CYTOTOXICITY | 0.0423 | 9 | 382 | 120 | 17890 | 3.51 (1.56 - 6.96) | 0 |
| GSEA_go_bp | G1_S_TRANSITION_OF_MITOTIC_CELL_CYCLE | 0.0444 | 4 | 387 | 23 | 17987 | 8.08 (2.02 - 23.82) | 0 |
| GSEA_go_bp | REGULATION_OF_DNA_BINDING | 0.0444 | 5 | 386 | 40 | 17970 | 5.82 (1.78 - 14.85) | 0 |
| GSEA_go_bp | POSITIVE_REGULATION_OF_MULTICELLULAR_ORGANISMAL_PROCESS | 0.0444 | 6 | 385 | 58 | 17952 | 4.82 (1.69 - 11.24) | 0,17 |
| GSEA_go_bp | POSITIVE_REGULATION_OF_BIOLOGICAL_PROCESS | 0.0444 | 27 | 364 | 656 | 17354 | 1.96 (1.27 - 2.93) | 0,11 |
| GSEA_go_bp | NEGATIVE_REGULATION_OF_CELLULAR_PROCESS | 0.0444 | 25 | 366 | 599 | 17411 | 1.99 (1.26 - 3.01) | 0,2 |
| GSEA_biocarta | BIOCARTA_CCR5_PATHWAY | 0.045 | 3 | 388 | 14 | 17996 | 9.94 (1.82 - 35.76) | 0 |
| GSEA_biocarta | BIOCARTA_MPR_PATHWAY | 0.045 | 4 | 387 | 29 | 17981 | 6.41 (1.63 - 18.37) | 0 |
| GSEA_canonical | PID_IL27_PATHWAY | 0.0464 | 4 | 387 | 22 | 17988 | 8.45 (2.11 - 25.04) | 0 |
| GSEA_canonical | PID_CXCR3_PATHWAY | 0.0464 | 5 | 386 | 38 | 17972 | 6.13 (1.87 - 15.7) | 0 |
| GSEA_canonical | REACTOME_CELL_SURFACE_INTERACTIONS_AT_THE_VASCULAR_WALL | 0.0464 | 7 | 384 | 77 | 17933 | 4.25 (1.64 - 9.26) | 0 |
| GSEA_canonical | REACTOME_G_ALPHA_I_SIGNALLING_EVENTS | 0.0464 | 11 | 380 | 174 | 17836 | 2.97 (1.44 - 5.5) | 0,18 |
| GSEA_canonical | SA_REG_CASCADE_OF_CYCLIN_EXPR | 0.0491 | 3 | 388 | 10 | 18000 | 13.92 (2.45 - 54.27) | 0 |
| GSEA_canonical | REACTOME_TRANSLOCATION_OF_ZAP_70_TO_IMMUNOLOGICAL_SYNAPSE | 0.0491 | 3 | 388 | 10 | 18000 | 13.92 (2.45 - 54.27) | 0 |

**Supplementary Table T5.** Associations between the presence of tertiary lymphoid structure (TLS) and the PET related features.

| **Characteristics** | **No TLS** | **Presence of TLS** | **P-value** |
| --- | --- | --- | --- |
| PC1 | -0.1045 ± 1.744 | 0.0778 ± 1.28 | 0.2750 |
| PC2 | 0.0181 ± 1.0396 | -0.2451 ± 0.5585 | 0.2390 |
| SUVmax | 14.0 ± 9.8 | 15.6 ± 8.2 | 0.3210 |
| SUVmean | 3.9 ± 2.7 | 4.7 ± 1.7 | 0.0633 |
| SUVpeak | 10.5 ± 7.8 | 11.3 ± 6.9 | 0.4660 |
| TLG | 1080.5 ± 1732.6 | 912.7 ± 1050.3 | 0.5250 |
| MTV | 138.6 ± 233.8 | 126.7 ± 112.1 | 0.3490 |

NOTE.- Tests are unpaired Wilcoxon tests. Data are given in each group as mean with with standard deviation.

**Supplementary Table T6.** Associations between cell densities for the 6 immunostainings performed and PCA outputs.

| **Cell density** |  | **Correlation with PC1** | |  | **Correlation with gene expression level** | | |  | **Average value** | |
| --- | --- | --- | --- | --- | --- | --- | --- | --- | --- | --- |
|  |  | **Spearman rho** | ***P*-value** |  | **Gene** | **Spearman rho** | ***P*-value** |  | **Metabolic-low** | **Metabolic-high** |
| **CD8** |  | 0.381 | **0.0354*** |  | CD8A | 0.788 | **<0.0001***** |  | 180.2 ± 272.8 | **258.6 ± 197.5** |
|  |  |  |  |  | CD8B | 0.722 | **<0.0001***** |  |  |  |
| **CD14** |  | 0.405 | **0.0247*** |  | CD14 | 0.489 | **0.0067*** |  | 1204.8 ± 1197.4 | **1743.5 ± 878.1** |
| **CD20** |  | 0.120 | **0.5190** |  | MS4A1 | 0.261 | 0.1629 |  | 50.3 ± 128 | 16.6 ± 18.6 |
| **CD45** |  | 0.383 | **0.0335*** |  | PTPRC | 0.627 | **0.0002***** |  | 594.7 ± 697.6 | **784.4 ± 688.9** |
| **CD68** |  | 0.371 | **0.0407*** |  | CD68 | 0.576 | **0.0011**** |  | 551.1 ± 724.1 | **642.9 ± 488.5** |
| **cMAF** |  | 0.356 | 0.0499* |  | c-MAF | 0.536 | **0.0026**** |  | 369.9 ± 460.1 | **378.4 ± 229** |

NOTE.-Tests are Spearman rank tests. *: P<0.05, **: P<0.005, ***: P<0.001. Data are given in each group as mean with with standard deviation.
